# Supplementary material for: The ‘wickedness’ of governing land subsidence: Policy perspectives from urban Southeast Asia
Source: PLoS One. 2021 Jun 9;16(6):e0250208. doi: 10.1371/journal.pone.0250208 (PMC8189459; doi:10.1371/journal.pone.0250208)
Supplement: Attachment — Submitted filename: Response to Reviewers.docx [file pone.0250208.s002.docx]

**Response to Editors and Reviewers – The “wickedness” of governing land subsidence: Policy perspectives from urban Southeast Asia**

**Editorial concerns**

1. We have checked the file templates and the following changes were made: a) to the titling in the manuscript, together with the appropriate line numbering, double spacing, and pagination (in keeping with the submission guidelines); b) the referencing has been changed to Vancouver style; c) all footnotes have been deleted
2. We have placed wickedness in inverted commas in order to draw attention to its policy peculiarity. A conceptual description has been given in section 1.3 of the paper.
3. Details regarding verbal participant consent have been provided in the ethics statement in the Methods and online submission information. No minors participated in the study.
4. The title page has now been uploaded as a separate file.
5. An ORCID ID has been created.
6. In the cover letter we have detailed our ethical restrictions in sharing our in-depth interview transcripts. However recent interview survey material gleaned in March 2020 has been uploaded.
7. Figures from the manuscript have been removed. Figs 1 and 2 have been uploaded in .tiff format.
8. Abstract has been added after the title page.
9. **On copyrights pertaining to the satellite images:** We used Copernicus and SRTM data in the figure. Copernicus data is copyright of ESA, but we can freely use them as long as we provide appropriate credit.
   We have thus added the following statement to the figure caption:
   "Contains Copernicus data 2018.”
   i. More information on Sentinel-1 data credit in the following links:
   <https://sentinel.esa.int/web/sentinel/terms-conditions>
   <https://scihub.copernicus.eu/twiki/pub/SciHubWebPortal/TermsConditions/TC_Sentinel_Data_31072014.pdf>
   SRTM data is public domain and can be used without any restriction:
   <https://catalog.data.gov/dataset/shuttle-radar-topography-mission-dted-level-1-3-arc-second-data-dted-1>
   We have also added the following SRTM publication in the references:

Farr, T. G., and Mike K. "Shuttle Radar Topography Mission produces a wealth of data." Eos, Transactions American Geophysical Union 81.48 (2000): 583-585.

10. Where relevant, captions for supporting information files have been provided at the end of the manuscript with updates for the in-text citation.

**Concerns raised by the reviewers:**

1. The last empirical section of the paper (Section 6) has been strengthened with recent fieldwork findings from Jakarta that were gleaned in March 2020.
2. To Reviewer 1 (R1): With regard to the data availability statement, we have provided anonymised quantified data for a recent social survey conducted in March 2020. The interview transcripts themselves are confidential as anonymity was guaranteed to all participants as their narratives/discussions were taped.
3. R1: A several paragraphs have been added on more recent policy development in Jakarta, including the Great Garuda megaproject. Please refer to the following lines: 569-575 and 650-671. We have touched upon Jakarta’s impending relocation plans elsewhere in the paper. However, for the sake of brevity, we have not delved into this in detail.
4. Reviewer 2 (R2): All long-winded sentences have been shortened and redrafted for clarity and brevity. These sections have been highlighted in yellow or marked out in the manuscript as tracked changes. We have also checked for repetitions and deleted them.
5. R2 (on geodesy data):

- We have added a new table (Table 1) to the paper. The newer map has been added (see Fig 1), replacing the former.
- The question regarding **reference points for constraining our maps:**
  To constrain the InSAR displacements, we used GNSS measurements in Jakarta and Manilla (Displayed by circles in Figure 1). In Jakarta, a GNSS station without significant displacement is used and in Manila, the vertical displacement from PIMO IGS permanent station is used to constrain the vertical displacement from InSAR. In Singapore, we did not use any external information and constrained the InSAR displacements by assuming the average of all InSAR observation points is zero.
- **With regard to the values in Singapore above the RMS:**

The RMS of displacement rate in Singapore is as small as 2.5 mm/a. There are areas with displacement rates higher than the estimated RMS. Because we did not have any absolute GNSS point in this area, we constrain the displacement rates by assuming the displacement rates average zero. Such assumption might slightly bias the estimated displacement rates. Therefore, small-scale displacements within the range of a few mm/a should be interpreted very carefully. In several reclamation areas, as discussed in the manuscript, the displacement rates can be reliably interpreted as land subsidence.

- **In order to explain the conversion from LOS to vertical:**

InSAR measures the displacement in satellite Line-of-Sight (LOS) direction which is defined by geometry of the sensor. Assuming the horizontal components of displacement are negligible, we use the incidence angle (θ) to convert the LOS measurement to vertical displacement V = LOS / cos(θ).

6. We have reformulated a number of ambiguous sentences that were highlighted, including part 6.3, and the point raised on inelastic compaction. We thank the Reviewer 2 for raising this point.
7. We have added to the introduction other subsiding regions around the world, while citing the papers that were shared by R2.
