# Supplementary material for: The ‘wickedness’ of governing land subsidence: Policy perspectives from urban Southeast Asia
Source: PLoS One. 2021 Jun 9;16(6):e0250208. doi: 10.1371/journal.pone.0250208 (PMC8189459; doi:10.1371/journal.pone.0250208)
Supplement: Attachment — Submitted filename: The comments1105.docx [file pone.0250208.s003.docx]

Taking Jakarta, Manila and Singapore as case studies, the author defines land subsidence as a typical "wicked" policy issue and reveals a broader coastal governance pattern. But some revisions are needed before they can be published. The comments are as follows:

（1）How to validate subsidence results of sentinel-1? The article lacks the necessary validation results, such as graphs and tables. Please refer to ‘Time-Series Evolution Patterns of Land Subsidence in the Eastern Beijing Plain, China’.

（2）Is sea level rise only for data reference? Is there a relationship between subsidence and sea level rise? Is the condition of subsidence and sea level interaction used in the analysis? If so, please indicate.

（3）Fig.1 is not professional and lacks the basic elements of the map, such as the compass, the marking of rapid subsidence area and the coverage of sentinel-1. Please refer to the papers on land subsidence, such as‘Land Subsidence Response to Different Land Use Types and Water Resource Utilization in Beijing-Tianjin-Hebei, China’.

（4）The geographical location and boundaries of the study area are shown in the form of maps.

（5）In line 339, "the subordination levels" is not a descriptive term for a sinking scientific paper.
